# Functional outcome and survival following spontaneous intracerebral hemorrhage: A retrospective population‐based study

**DOI:** 10.1002/brb3.1113

**Published:** 2018-09-21

**Authors:** Lise R. Øie, Mattis A. Madsbu, Ole Solheim, Asgeir S. Jakola, Charalampis Giannadakis, Anders Vorhaug, Llewellyn Padayachy, Heidi Jensberg, David Dodick, Øyvind Salvesen, Sasha Gulati

**Affiliations:** ^1^ Department of Neurology St Olavs Hospital, Trondheim University Hospital Trondheim Norway; ^2^ Department of Neuromedicine and Movement Science, Faculty of Medicine and Health Sciences Norwegian University of Science and Technology (NTNU) Trondheim Norway; ^3^ Department of Neurosurgery St. Olavs hospital, Trondheim University Hospital Trondheim Norway; ^4^ Department of Neurosurgery Sahlgrenska University Hospital Gothenburg Sweden; ^5^ Institute of Neuroscience and Physiology University of Gothenburg, Sahlgrenska Academy Gothenburg Sweden; ^6^ Department of Neurosurgery University of Cape Town Cape Town South Africa; ^7^ Department of Health Registries Trondheim Norway; ^8^ Department of Neurology Mayo Clinic Phoenix Arizona; ^9^ Department of Public Health and General Practice Norwegian University of Science and Technology (NTNU) Trondheim Norway

**Keywords:** intracerebral hemorrhage, outcome, population‐based, predictors, survival

## Abstract

**Background:**

Accurate and reliable clinical and radiological predictors of intracerebral hemorrhage (ICH) outcomes are needed to optimize treatment of ICH. The aim of this study was to investigate functional outcome and identify predictors of severe disability or death following ICH.

**Materials and methods:**

Retrospective population‐based study of spontaneous ICH. Clinical and radiological data were obtained from electronic medical records, and functional outcome estimated using the modified Rankin Scale (mRS) before ICH and at 3 and 12 months after ICH.

**Results:**

Four hundred and fifty‐two patients were included (mean age 74.8 years, 45.6% females). Proportion of fatal outcome at 1 week was 22.1%, at 3 months 39.2%, and at 12 months 44.9%. Median mRS score before the ICH was 1 (interquartile range [IQR] 2); for survivors at 3 months, it was 5 (IQR 3); and at 12 months, it was 3 (IQR 2). Independent predictors of severe disability (mRS of 5) or death (mRS of 6) were use of oral antithrombotic drugs (OR 2.2, 95% CI 1.3–3.8, *p* = 0.04), mRS score before the ICH (OR 1.8, 95% CI 1.4–2.2, *p* < 0.001), Glasgow Coma Scale (GCS) on admission (OR 8.3, 95% CI 3.5–19.7, *p* < 0.001), hematoma volume >60 ml (OR 4.5, 05% CI 2.0–10.2, *p* < 0.001), and intraventricular hematoma extension (OR 1.8, 95% CI 0.8–4.2, *p* < 0.001).

**Conclusion:**

Intracerebral hemorrhage is associated with high mortality, and more than one third of survivors end up with severe disability or death 3 months later. Predictors of severe disability or death were use of oral antithrombotic drugs, functional disability prior to ICH, low GCS on admission, larger hematoma volume, and intraventricular hematoma extension.

## 1 INTRODUCTION

Spontaneous intracerebral hemorrhage (ICH) accounts for approximately 10% to 15% of all cases of stroke, but is generally associated with higher risk of death and greater loss of health over a lifetime than ischemic stroke (Cadilhac, Dewey, Vos, Carter, & Thrift, 2010; Lee, Hwang, Jeng, & Wang, 2010; van Asch et al., 2010). Despite the severe and devastating consequences of ICH, optimal management is still under discussion. To optimize treatment strategies, accurate and reliable clinical and radiological ICH outcome predictors are needed.

Previous studies on outcomes following ICH are often either limited in size or carried out in selected patients referred to major hospitals. Between 25%–50% of patients with ICH die within the first month (Qureshi et al., 2001), and only 20% regain functional independency at 6 months (Flemming, Wijdicks, & Li, 2001). Several grading systems for predicting ICH mortality exist, using different clinical and diagnostic imaging characteristics (Hemphill, Bonovich, Besmertis, Manley, & Johnston, 2001; Rost et al., 2008; Ruiz‐Sandoval, Chiquete, Romero‐Vargas, Padilla‐Martinez, & Gonzalez‐Cornejo, 2007). Level of consciousness and baseline volume of parenchymal hemorrhage have been shown to be independent predictors of poor outcome (Davis et al., 2006; Flemming et al., 2001; Nilsson, Lindgren, Brandt, & Saveland, 2002; Safatli et al., 2016), whereas the impact of intraventricular extension and hematoma location remains more uncertain (Bhatia et al., 2013; Flemming et al., 2001). The goal of this population‐based study was to investigate functional outcome in patients with ICH in a high resource setting. We also sought to identify predictors of severe disability or death following ICH.

## 2 MATERIALS AND METHODS

### 2.1 Ethical approval

The Regional Committee for Medical and Health Research Ethics in Central Norway approved the study (2014/958) and waived the requirement of informed consent.

### 2.2 The Norwegian health care system

Norway has a public health care system. Acute illness requiring hospital admission is treated free of charge by the public health care and insurance policies do not influence the diagnosis or treatment of ICH. Only public hospitals provide health care to patients with ICH, and the health authorities cover all inpatient treatment.

### 2.3 Norwegian patient registry

Norwegian patient registry (NRF) is a national administrative health registry containing person identifiable information on all inpatient and outpatient treatment by the public Norwegian specialist health care services. The database contains demographic, administrative, and health related data, such as dates of admission and discharge, and primary and unlimited number of secondary diagnoses according to the 10th revision of the International Classification of Diseases (ICD‐10), including codes for diagnostic and therapeutic procedures. All discharge diagnoses are exclusively assigned by the physicians treating the patient, and cannot later be altered. The registry receives data monthly and is used for reimbursement purposes, hospital activity statistics, and research (Varmdal et al., 2016). Coding of ICH discharge diagnoses in the NPR is of high quality, with positive predictive values for ICH hospital admissions exceeding 95% (Oie et al., 2018).

### 2.4 Study population

The study was carried out within the population of Sør‐Trøndelag County between 1 January 2008 and 31 December 2014. The study population of 298,000 individuals (2009 Census, Statistics Norway) is served by St. Olavs University Hospital, the only hospital in this defined geographical region providing inpatient treatment of ICH. Patients with a primary diagnosis of spontaneous ICH (ICD‐10 codes I61.0–I61.9) and a residential address within Sør‐Trøndelag County were included in the study. After review of hospital records and diagnostic images, we excluded traumatic ICH, ICH related to intracranial tumors, extra‐axial intracranial hemorrhages, and ICH related to parenteral thrombolytic treatment (inpatient ICH). Patients with isolated intraventricular hemorrhage were included. Patients whose records were not found, or episodes of ICH occurring outside the study period were excluded from the analyses. Patients from the study area who were hospitalized elsewhere for ICH and subsequently transferred to St. Olavs University hospital were included. All included patients were aged 18 years or older.

### 2.5 Baseline recordings

ICH was defined as clinical symptoms of stroke combined with the presence of parenchymal hemorrhage on a cerebral CT (cCT) scan. Time of ictus was defined as the time when neurological symptoms appeared. Demographic data, comorbidities, clinical and radiological data, and information about surgical interventions were retrieved from the electronic medical records. The Charlson comorbidity index was used to estimate comorbidity, a scoring system predicting 10‐year survival in patients with multiple comorbidities. In addition to vascular comorbidities, it includes dementia and malignancy which are often not included in other scoring systems (Charlson, Pompei, Ales, & MacKenzie, 1987; Sundararajan et al., 2004).

Use of oral antithrombotic medications (OAM) at the time of hospital admission was also registered, including both antiplatelet agents (aspirin, dipyridamole, acetylsalicylic acid‐dypiridamole, and clopidogrel) and anticoagulant agents (warfarin, dabigatran, apixaban, and rivaroxaban).

A baseline cCT scan was available for all included patients, except for patients transferred from hospitals outside Norway. Four of the authors, blinded to patient characteristics, assessed all cCTs. Hematoma location was classified as lobar (cortical or subcortical white matter), deep (thalamus and basal ganglia), brainstem, or cerebellum. Hematoma volume was calculated manually with the formula A × B × C /2 cm^3^ (Kothari et al., 1996). The presence of intraventricular hemorrhage was registered, and a modified Graeb score (mGS) was calculated for intraventricular hemorrhages (Hinson, Hanley, & Ziai, 2010). Hematoma expansion was determined if sequential brain imaging was available, and defined as a relative parenchymal volume increase of more than 33% from initial to follow‐up imaging within 3 to 72 hr (Kuramatsu et al., 2015). If more than one follow‐up imaging session was performed, the one closest to the 24‐hr time was chosen. Patients with hematoma evacuation before any follow‐up imaging were excluded from the hematoma expansion analysis.

### 2.6 Outcome assessment

All included patients were followed up for a minimum of 12 months. Level of consciousness, determined by Glasgow Coma Scale (GCS), is routinely scored for all patients admitted to the hospital, and was found in the electronic medical records. Functional outcome was determined using the modified Rankin Scale (mRS) score before the ICH, and at 3 and 12 months after ICH. The mRS score prior to and immediately after the ICH is routinely scored on admission to the stroke unit by stroke physicians, nurses, and physiotherapists. Three months after discharge, stroke patients receive either an outpatient or home visit where mRS is usually scored (Indredavik, Fjaertoft, Ekeberg, Loge, & Morch, 2000). This information is available in the electronic medical records. The mRS scores at 12 months were scored by two of the authors (LRØ, MAM) based on information from the electronic medical records. Patients with mRS of 5 were classified as having severe disability and mRS 6 as dead. As an additional indicator for clinical outcome, cohabitation before and after the ICH was assessed. The date of death was provided by either the electronic medical record or Norwegian Institute of Public Health.

## 3 STATISTICS

Statistical analyses were performed with SPSS 24.0. Descriptive statistics were computed for baseline characteristics. To compare users and nonusers of OAM, we used independent samples *t* tests for continuous variables, and chi‐square test for categorical variables. Predictors of severe disability or death (mRS 5 and 6) at 3 months were analyzed using Cox regression analyses. Variables from the univariable analyses with a *p*‐value <0.10 were included in the multivariable model. Effect sizes were presented as odds ratio with 95% confidence intervals. *p*‐Values <0.05 were considered statistically significant. Survival analyses of patients younger or older than 75 years and users and nonusers of OAM were analyzed with log‐rank tests and visualized using Kaplan–Meier curves. Aggregated exposure for inhabitants of Sør‐Trøndelag County between 1 January 2008 and 31 December 2014 was used to calculate the crude incidence rate.

## 4 RESULTS

In total, 561 patients with spontaneous ICH requiring hospitalization were identified in the NPR within the study period, and based on our inclusion and exclusion criteria, 452 patients were included for further analyses.

### 4.1 Patient characteristics

Crude annual incidence rate between 2008 and 2014 in Sør‐Trøndelag County was 21.5 per 100,000 per year. Patient and hematoma characteristics are presented in Table 1. The mean age was 74.8 years and 45.6% were females. Past history of arterial hypertension was present in 52.7% (*n* = 238) and a history of diabetes in 11.7% (*n* = 53). The mean Charlson comorbidity index score was 1.4 (*SD* 1.6). At the time of the ICH, a total of 56.6% (*n* = 256) used OAM, with antiplatelet drugs (37.2%) being more frequently used than anticoagulants (22.8%). 14 patients (5.5%) used both antiplatelets and anticoagulants at the time of ICH. Novel oral anticoagulants (NOAC) were introduced in the last part of the inclusion period, and only 1 patient used NOAC (rivaroxaban), whereas the others used warfarin with a mean INR value of 2.8 on admission. As presented in Table 4, users of OAM were older than nonusers (78.6 vs. 69.4 years, mean difference 9.2 years, 95% CI 7.0–11.3, *p* < 0.001). In total, 9.5% (*n* = 43) underwent early surgical treatment for the bleeding. Of these, 6.2% (*n* = 28) underwent hematoma evacuation, and 0.7% (*n* = 3) received an additional craniectomy (Table 1).

**Table 1:** Patient and hematoma characteristics

	Total, *N*, (%) *N* = 452 (100)	Missing data
Demographic		
Female sex, *N* (%)	206 (45.6)	
Age, mean, ±*SD*	74.8 ± 12.1	
Age, median (IQR, range)	78 (17, 22–96)	
Age ≥76 years	256 (56.6)	
Current smoking	73 (16.2)	43 (9.5)
Cohabitation		
Before ICH		
Home	314 (69.5)	
Home with assistance	67 (14.8)	
Assisted facility	22 (4.9)	
Nursing home	49 (10.8)	
After ICH		
Home	83 (18.4)	
Home with assistance	32 (7.1)	
Assisted facility	73 (16.2)	
Nursing home	122 (27.0)	
Death during acute stay	142 (31.4)	
Comorbidities	398 (88.1)	
Hypertension	238 (52.7)	
Atrial fibrillation	99 (21.9)	
Diabetes mellitus	53 (11.7)	
Uncomplicated	50 (11.5)	
End organ failure	3 (0.7)	
Hyperlipidemia	71 (15.7)	
Ischemic heart disease	99 (21.9)	
Congestive heart disease	41 (9.1)	
Previous ischemic stroke	112 (24.8)	
Previous hemorrhagic stroke	28 (6.2)	
Intracerebral hemorrhage	16 (3.5)	
Subdural hemorrhage	3 (0.7)	
Subarachnoid hemorrhage	9 (2.0)	
Epidural hemorrhage	1 (0.2)	
Venous thromboembolism	22 (4.9)	
Peripheral vascular disease	14 (3.1)	
Dementia	50 (11.1)	
Renal failure	12 (2.6)	10 (2.2)
GFR >60 ml	6 (1.3)	
GFR <60 ml	6 (1.3)	
CODP	40 (8.8)	
Connective tissue disease	12 (2.7)	
Peptic ulcer disease	29 (6.4)	
Liver disease	7 (1.4)	
Mild	2 (0.4)	
Moderate/Severe	5 (1.1)	
Hemiplegia	8 (1.8)	
Malignant lymphoma	4 (0.9)	
Solid tumor	23 (5.1)	
Metastatic tumor	9 (2.0)	
Charlson comorbidity index		
Mean (*SD*)	1.4 (1.6)	
Oral antithrombotic drugs	256 (56.6)	
Antiplatelets	168 (37.2)	11 (2.4)
Aspirin	165 (36.5)	
Dipyridamole	15 (3.3)	
Dipyridamole + ASA	1 (0.2)	
Clopidogrel	7 (1.5)	
Anticoagulants	103 (22.8)	10 (2.2)
Warfarin	102 (22.6)	
INR value, mean (*SD*)	2.8 (1.3)	3 (2.9)
INR >3, mean volume	22.7 (30.7)	
Rivaroxaban	1 (0.2)	
Surgical management	43 (9.5)	
Intracranial pressure monitoring	8 (1.8)	
Hematoma evacuation	28 (6.2)	
External ventricular drainage	29 (6.4)	
Hemicraniectomy	3 (0.7)	
Hematoma characteristics, initial CT		
Location	451 (99.8)	1 (0.2)
Lobar	185 (40.9)	
Deep	203 (44.9)	
Brainstem	23 (5.1)	
Cerebellum	37 (8.2)	
Strict IVH	3 (0.7)	
Mean volume (*SD*)	33.1 (45.7)	5 (1.1)
<30 ml	298 (65.9)	
30–60 ml	67 (14.8)	
>60 ml	82 (18.1)	
Intraventricular hemorrhage	186 (41.2)	
Graeb score,		
Mean (*SD*)	11.7 (8.1)	
Median (IQR, range)	11 (14, 0–30)	
Hematoma characteristics, follow‐up CT		
Expansion	45 (10.2)	
Modified Graeb score		
Mean (*SD*)	8.4 (8.3)	
Median (IQR)	7 (16, 0–24)	

### 4.2 Radiological characteristics

The most common location of the hemorrhage was deep (44.9%, *n* = 203), followed by lobar (40.9%, *n* = 185), cerebellar (8.2%, *n* = 37), brainstem (5.1%, *n* = 23), and strict intraventricular hemorrhage (0.7%, *n* = 3) (Table 1). Among patients with supratentorial ICH, the prevalence of hypertension was higher in patients with deep location (59.5%) compared to lobar location (50.5%) (*p* = 0.005). Overall, the mean baseline hematoma volume was 33.1 ml (*SD* 45.7). Intraventricular hemorrhage was observed in 186 patients (41.2%) and hematoma expansion in 46 patients (10.2%). Mean hematoma volume of users of OAM (Table 4) was higher than for nonusers (35.8 vs. 26.3 ml, mean difference 9.5 ml, 95% CI 1.2–17.8, *p* = 0.025).

### 4.3 Functional outcome

**Figure 1:**
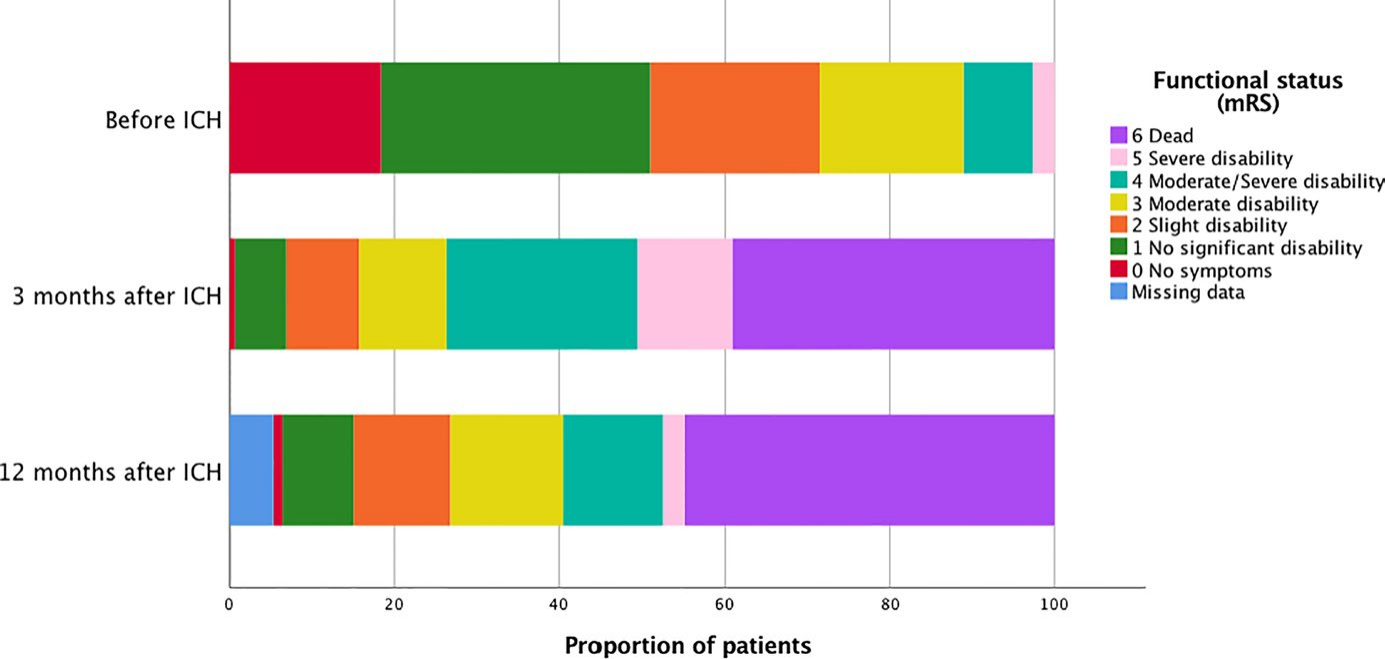
Functional status (mRS)

Clinical presentation and functional outcome are presented in Table 2. Median GCS score on admission was 14 (IQR 6). Median mRS score before the ICH was 1 (IQR 2); for survivors at 3 months, the median mRS was 5 (IQR 3); and at 12 months, median mRS was 3 (IQR 2). The proportion of fatal outcome during the first week was 22.1% (100 of 452 patients), at 3 months 39.2% (177/452), and at 12 months 44.9% (203/452). Patients who survived the ICH at 3 months were younger than the ones who died (78.6 vs. 72.4 years, mean difference 6.2 years, 95% CI 1.1–4.0, *p* < 0.001). Among 275 survivors at 3 months, 52 (18.9%) were severe disabled (mRS 5), and at 12 months, 12 (4.8%) had mRS 5. Functional status before, and at 3 and 12 months is presented in Figure 1.

**Table 2:** Clinical presentation

Clinical presentation	No (%)	Missing data
Before ICH		
Median mRS score (IQR, range)	1 (2, 0–5)	
Severe disability (mRS 5)	12 (2.7)	
On admission		
GCS score on admission		
Median GCS (IQR, range)	14 (6, 3–15)	
9–15	344 (76.1)	
3–8	108 (23.9)	
Follow‐up		
1 week		
Fatal outcome within 1 week	100 (22.1)	
3 months		
Overall median mRS (IQR, range)	5 (3, 0–6)	
Severe disability (mRS 5)	52 (18.9)	
Fatal outcome (mRS 6)	177 (39.2)	
Median mRS (IQR, range) for survivors	4 (2, 0–5)	
12 months		24 (5.3)
Overall median mRS (IQR, range)	5 (3, 0–6)	
Severe disability (mRS 5)	12 (4.8)	
Fatal outcome (mRS 6)	203 (44.9)	
Median mRS (IQR, range) for survivors	3 (2,0–5)	

The univariate analyses (Table 3) showed the following variables as significant predictors of severe disability or death within 3 months: age ≥75 years (OR 2.8, 95% CI 1.9–4.2, *p* < 0.001), increasing Charlson comorbidity index score (OR 1.3, 95% CI 1.1–1.5, *p* < 0.001), OAM (OR 2.9, 95% CI 1.9–4.2, *p* < 0.001), high mRS score before stroke (OR 1.9, 95% CI 1.6–2.3, *p* < 0.001), GCS <9 on admission (OR 20.8, 95% CI 9.8–44.2, *p* < 0.001), hematoma volume >60 ml (OR 7.2, 95% CI 3.8–13.4, *p* < 0.001), intraventricular hemorrhage (OR 6.5, 95% CI 4.2–9.8, *p* < 0.001), and hydrocephalus (OR 9.2, 95% CI 4.8–17.4, *p* < 0.001). In the multivariable analysis, use of OAM (OR 2.2, 95% CI 1.3–3.8, *p* = 0.04), high mRS before stroke (OR 1.8, 95% CI 1.4–2.2, *p* < 0.001), GCS<9 on admission (OR 8.3, 95% CI 3.5–19.7, *p* < 0.001), hematoma volume >60 ml (OR 4.5, 05% CI 2.0–10.2, *p* < 0.001), and intraventricular hemorrhage (OR 1.8, 95% CI 0.8–4.2, *p* < 0.001) remained independent predictors of severe disability or death 3 months after the ICH. In a similar multivariate analysis, only age <75 years was found to be an independent predictor of favorable outcome defined as mRS 0–2 at three months (OR 0.2, 95% CI 0.1–0.3, *p* < 0.001).

**Table 3:** Factors associated with severe disability or death (mRS score 5–6) at 3‐month follow‐up

Variable	Univariable regression		Multivariable regression	
	OR (95% CI)	*p*‐Value	OR (95% CI)	*p*‐Value
Age >75 years	2.8 (1.9–4.2)	<0.001	1.5 (0.9–2.6)	0.135
Female sex	1.3 (0.9–1.8)	0.211	—	—
Charlson comorbidity index^a^	1.3 (1.1–1.5)	<0.001	1.1 (0.9–1.3)	0.380
Oral antithrombotic drugs	2.9 (1.9–4.2)	<0.001	2.2 (1.3–3.8)	0.004
mRS score before stroke^b^	1.9 (1.6–2.3)	<0.001	1.8 (1.4–2.2)	<0.001
GCS <9 on admission	20.8 (9.8–44.2)	<0.001	8.3 (3.5–19.7)	<0.001
Infratentorial location	1.3 (0.8–2.3)	0.328	—	—
Hematoma volume >60 ml	7.2 (3.8–13.4)	<0.001	4.5 (2.0–10.2)	<0.001
Intraventricular hemorrhage	6.5 (4.2–9.8)	<0.001	3.5 (2.0–6.2)	<0.001
Hydrocephalus	9.2 (4.8–17.4)	<0.001	1.8 (0.8–4.2)	0.152

a Per increment.

b Score range from mRS 0 (no symptoms) to 6 (death) (per increment).

**Figure 2:**
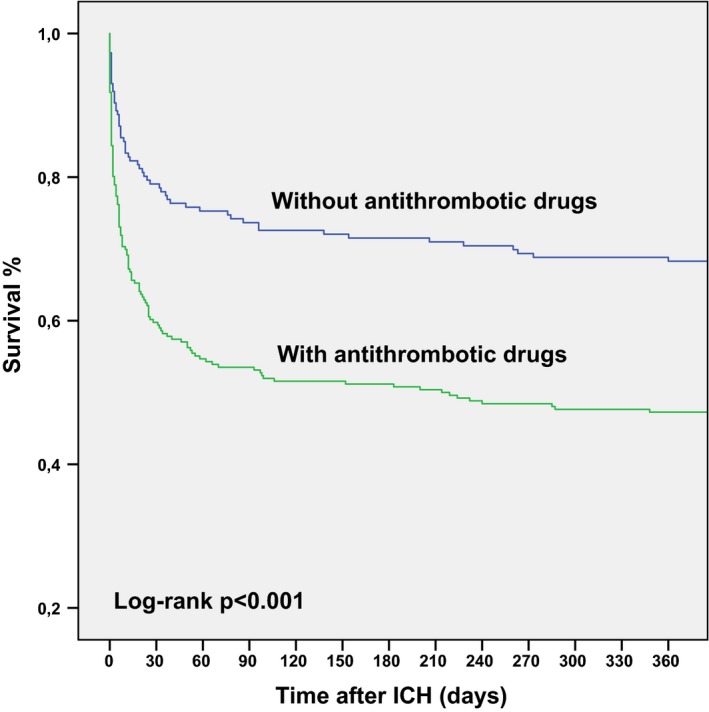
Survival curve for users and nonusers of oral antithrombotic medications

Use of OAM was strongly associated with an increased mortality at 3 months (Figure 2). Among those using OAM, 46.5% (119 of 256 patients) were dead at 3 months, compared to 26.3% (49 of 186) in nonusers (*p* < 0.001) (Table 4).

**Table 4:** ICH characteristics in users versus nonusers of oral antithrombotic drugs

Variables	Users	Nonusers	*p*‐Value (95% CI for mean difference)
Age (years)	78.6 (9.1)	69.4 (13.7)	<0.001 (7.0–11.3)
Volume on admission (ml)	35.8 (46.6)	26.3 (2.9)	0.025 (1.2–17.8)
Hematoma growth	24 (13.3)	12 (7.9)	0.116
Largest measured volume (ml)	38.3 (47.5)	27.8 (40.3)	0.015 (2.1–19.0)
Intraventricular hemorrhage	110 (43.0)	70 (37.6)	0.260
Initial modified Graeb Score, mean (*SD*)	12.4 (8.3)	10.6 (7.7)	0.162 (−0.7–4.2)
GCS on admission, mean	11.5 (4.2)	12.3 (3.8)	0.042 (0.03–1.6)
mRS before ICH, mean (*SD*)	2.0 (1.3)	1.3 (1.3)	<0.001 (0.4–0.9)
mRS at 3 months of survivors, mean (*SD*)	3.7 (1.1)	3.1 (1.3)	<0.001
Severe disability or death (mRS 5 + 6) at 3 months	155 (60.5)	65 (34.9)	<0.001
Severe disability or death (mRS 5 + 6) at 12 months	141 (58.0)	65 (37.1)	<0.001
Severe disability (mRS 5) at 3 months	36 (26.3)	16 (11.7)	0.003
Severe disability (mRS 5) at 12 months	6 (5.6)	6 (5.2)	1.000
3 months mortality (mRS 6)	119 (46.5)	49 (26.3)	<0.001
12 months mortality (mRS 12)	135 (52.7)	59 (31.7)	<0.001

Out of the 28 patients that underwent surgical hematoma evacuation, 20 (71.4%) patients had a supratentorial location and 9 (32.1%) an infratentorial location. At 3 months, 2 (4.2%) patients had mRS 3, 10 (52.6%) patients had mRS 4, 7 (36.8%) patients had mRS 5 (severe disability), and 9 (32.1%) patients were dead (mRS 6). We were unable to score mRS for 24 (5.3%) patients at 12 months, as their medical records were missing. However, they were not registered as dead in the Norwegian Institute of Public Health; thus, we know they were alive.

Prior to the ICH, 314 (69.5%) of the patients lived at home. Three months later, only 83 (26.4%) of these continued living at home. Patients who survived the acute hospital stay and were discharged to their homes had significantly smaller hematoma volume than those who were discharged to a nursing home (mean difference 14.4 ml, 95% CI 9.0–19.8, *p* < 0.001).

## 5 DISCUSSION

This retrospective population‐based study in a high resource setting shows that ICH is associated with high mortality, and the majority of survivors become dependent of care.

Independent predictors of severe disability or death at three months were use of OAM, functional disability prior to the ICH, low GCS score on admission, larger hematoma volume, and presence of intraventricular extension. Only age <75 years was an independent predictor of favorable outcome at three months.

The mortality rate in the present study was 22.1% the first week, 39.2% at 3 months, and 44.9% at 12 months, which is in general agreement with data from previous studies, reporting mortality rates from 25%–50% (Flaherty et al., 2006; Gonzalez‐Perez, Gaist, Wallander, McFeat, & Garcia‐Rodriguez, 2013; Qureshi et al., 2001; Sacco, Marini, Toni, Olivieri, & Carolei, 2009). At 3 months, only 15.7% of the patients had a favorable outcome (mRS <2), correlating well with studies presenting functional independency rates from 12% to 39% (Flemming et al., 2001; Safatli et al., 2016; van Asch et al., 2010). However, it is difficult to compare results across studies due to variation in inclusion criteria, outcome measures, and statistical analyses.

In line with previous studies, we found that the patients’ initial level of consciousness, baseline hematoma volume, hematoma growth, hydrocephalus, and intraventricular extension were the most important prognostic factors of ICH (Bhatia et al., 2013; Davis et al., 2006; Flemming et al., 2001; Nilsson et al., 2002; Safatli et al., 2016; Sarker et al., 2008). Given the association of poor outcome with hematoma expansion, accurate and reliable predictors of expansion are needed. In contrast to volume and location, secondary hematoma growth is potentially modifiable, and prevention of such expansion should still be a major therapeutic target in the management of ICH. The spot sign observed on CT angiography (CTA) has been found to independently predict poor outcome (Demchuk et al., 2012). We did not evaluate the spot sign in our study as CT angiography was only available for some of the included patients. Predictors of hematoma growth on noncontrast computed tomography, such as the blend sign and hypodensities, have been found to predict outcome in patients with ICH (Sporns, Kemmling, Minnerup, Hanning, & Heindel, 2018). The major advantage of these features is greater availability in clinical practice, and also, they may serve as an alternative to CTA in the case of contrast allergy or renal dysfunction. Magnetic resonance imaging (MRI) may also predict hematoma volume and extension by detecting markers of cerebral small vessel disease (Boulouis et al., 2016). Finally, perihemorrhagic edema has been found to be an independent predictor of functional outcome after ICH (Volbers et al., 2018).

Incidence of ICH is increasing with age for both genders; however, consistent with previously published studies, neither gender nor age were significant predictors of severe outcome in our study (Broderick et al., 2007; Safatli et al., 2016). Conflicting results have been reported for hematoma location, and some studies have found infratentorial location to be associated with increased mortality (Safatli et al., 2016), while others failed to show an association (Bhatia et al., 2013). In the present study, hematoma location was not an independent prognostic factor of severe disability or death.

The proportion of patients with coexisting medical conditions reflects a generally high prevalence of atherosclerotic vascular disease among patients with spontaneous ICH. More than half of the patients had a history of hypertension, and 1/3 had experienced a cerebrovascular event prior to the ICH in our study. There was no statistically significant association between the 3‐month outcome and the presence of comorbidities. Nevertheless, we believe that primary prevention should still remain an important target in the management of ICH.

Overall, we found that more than fifty percent of the ICH were associated with OAM, with antiplatelets being more frequently used than anticoagulants (37.2% vs. 22.8%, respectively). Users of OAM were older and had poorer survival than nonusers. The current use of OAM was an independent predictor of severe disability or death at 3 months. The higher mortality found among patients receiving anticoagulants in our study is consistent with prior studies (Flibotte, Hagan, O'Donnell, Greenberg, & Rosand, 2004; Horstmann et al., 2013). NOAC‐associated ICH is associated with high mortality and unfavorable outcomes (Purrucker et al., 2016); however, when compared with warfarin, they were found to be associated with lower risk of in‐hospital mortality (Inohara et al., 2018). NOACs were introduced in the last part of our study, and only one of our included patients used a NOAC. Although aspirin is associated with lower risk of ICH (Garcia‐Rodriguez, Gaist, Morton, Cookson, & Gonzalez‐Perez, 2013), previous use of combination antiplatelet therapy in patients with ICH is associated with higher risk of in‐hospital mortality (Khan et al., 2017). Due to differences in study periods, patient populations, and OAM under investigation, it is challenging to compare the effect of OAM on clinical outcomes following ICH across studies. More results from pharmacoepidemiological studies are pending and may provide better real‐world estimates for risk of ICH in users of antithrombotic medications (Gulati et al., 2015).

Patients who survive ICH may have risk factors for future thromboembolic events, but the role of OAM remains a therapeutic dilemma with conflicting evidence and contradictory recommendations (Biffi et al., 2017; Pennlert et al., 2015). There is currently a lack of solid evidence to guide decisions on whether and when to start or restart treatment in ICH survivors, and both well designed randomized controlled trials and observational studies should be encouraged (Perry et al., 2017).

In the present cohort, patients who underwent neurosurgical interventions did not exhibit better outcome. At 3 months, more than one third of the patients receiving surgical management were dead (mRS 6), and another one third had severe disability (mRS 5). Our study was not a treatment trial, and was not designed to evaluate the effectiveness of surgical treatment of ICH. More severely affected patients are more likely to undergo surgical treatment, and thus, selection bias may have influenced the functional outcomes following surgical treatment in our study. However, two randomized controlled trials (Mendelow et al., 2005, 2013 ) failed to show benefit from early surgery when compared with conservative treatment. Still, only patients where the surgical indication was felt to be relative (i.e., clinical equipoise) were recruited in these studies.

### 5.1 Study strengths and limitations

The major strength of this study is the relatively large sample size, including all registered cases of ICH from a defined geographic area, served by one hospital. Patients with an ICH are treated in public hospitals with uniform access to high levels of care, and social and economic factors are less likely to influence outcome. Results are strengthened by the review of all diagnostic imaging and validation of diagnoses. However, a retrospective evaluation is challenging as medical records often lack important information. We were unable to determine functional outcome (mRS) 12 months after the ICH for 24 patients. The most likely explanation is that these patients either moved abroad where their medical records were no longer available, or they did not suffer from severe sequela after the ICH and thus did not seek medical care after the 3‐month follow‐up. Still, the number of patients without a medical record 12 months after ICH is relatively low and is unlikely to influence our results.

## 6 CONCLUSION

This retrospective population‐based study shows that ICH is associated with high mortality, and the majority of survivors become dependent of care. Independent predictors of severe disability or death at 3 months were use of oral antithrombotic drugs, functional disability prior to the ICH, low GCS on admission, larger hematoma volume, and intraventricular extension.
